# Prolapse of the Small Intestine from the Uterine Perforation at Dilatation and Curettage

**DOI:** 10.1155/2014/164356

**Published:** 2014-03-04

**Authors:** Shigeki Matsubara, Akihide Ohkuchi, Hiroaki Nonaka, Homare Ito, Alan T. Lefor

**Affiliations:** ^1^Department of Obstetrics and Gynecology, Jichi Medical University, 3311-1 Yakushiji, Shimotsuke, Tochigi 329-0498, Japan; ^2^Department of Surgery, Jichi Medical University, 3311-1 Yakushiji, Shimotsuke, Tochigi 329-0498, Japan

## Abstract

Dilatation and curettage (D&C) sometimes causes uterine perforation, which usually does not cause a serious problem. Here, we report uterine perforation caused by D&C, in which the small intestine prolapsed from the uterus, requiring intestinal resection. D&C was performed for missed abortion at 9 weeks. After dilating the cervix, forceps grasped tissue that, upon being pulled, resulted in the intestine being prolapsed into the vagina. Laparotomy revealed a perforation at the low anterior uterine wall, through which the ileum had prolapsed. The mesentery of the prolapsed ileum was completely detached and the ileum was necrotic, which was resected. The uterus and the intestine were reconstructed. Although intestinal prolapse is considered to be caused by “unsafe” D&C performed by inexperienced persons or even by nonphysicians in developing countries, this occurred in a tertiary center of a developed country. We must be aware that adverse events such as uterine perforation with intestinal prolapse can occur even during routine D&C.

## 1. Introduction

Conservative management is usually recommended for uterine perforation during dilatation and curettage (D&C); however, according to *Williams Obstetrics* Textbook [1] “considerable intra-abdominal damage can be caused by instrument passed through a uterine defect.” We here report a patient in whom the small intestine prolapsed through a uterine perforation to the vagina. Small intestinal mesentery was detached from the intestine, causing intestinal necrosis and requiring intestinal resection.

## 2. Case Presentation

**Figure 1:**
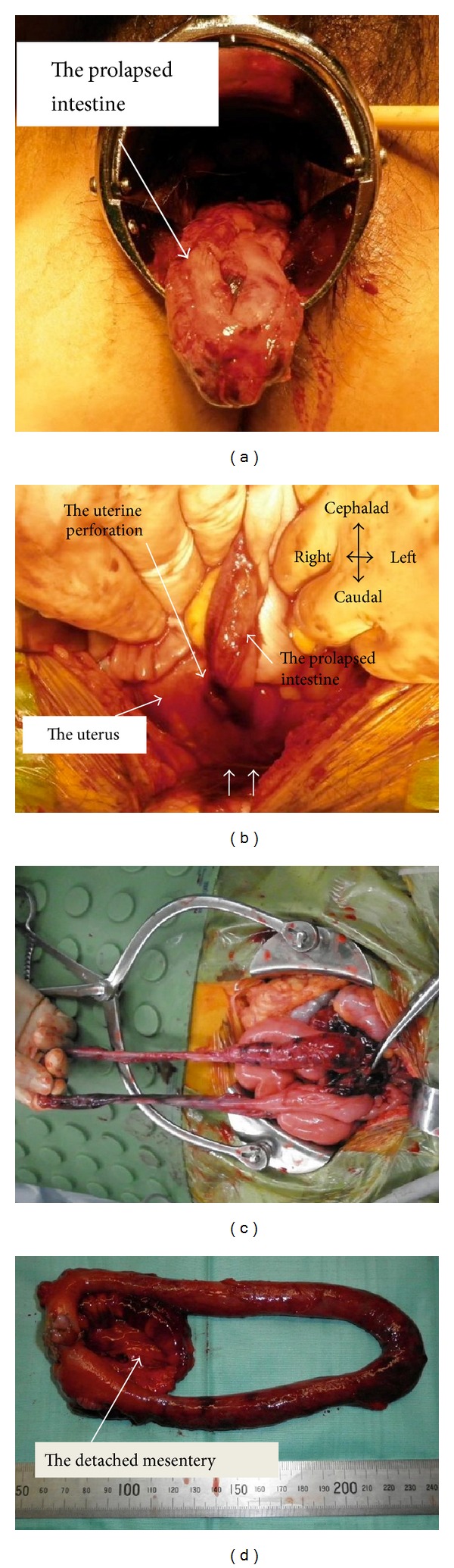
Prolapsed small intestine and operative findings. (a) The small intestine is observed in the vagina. (b) The ileum is prolapsed through the uterine perforation and somewhat retracted. The uterine fundus is not seen, hidden behind the operators' hands. Small arrows show the previous cesarean scar site, indicating that the perforation did not occur at the previous cesarean incision. The direction of the perforation is not distinguishable in this figure: it is illustrated in Figure 2. (c) The prolapsed intestine is necrotic. The tip of the forceps indicates the detached mesentery. (d) The resected specimen, showing that the mesentery (50 cm) was completely separated from the prolapsed ileum.

A D&C was performed on a 36-year-old 2 parous woman because of missed abortion at 9 weeks of gestation. She had undergone lower segment cesarean section twice. A gestational sac (GS) 34 mm in diameter with a 3 mm beatless embryo was observed within the uterine body, which was in slight anteversion and anteflexion. With a hygroscopic dilator placed for 12 hours, D&C was performed. Although abdominal ultrasound did not clearly show the sound, the procedure continued, expecting an “easy” D&C. The cervix was dilated with metal cervical dilator without difficulty. We usually use forceps and not a suction curette. We inserted the forceps into the uterine cavity, held the expected gestational sac, but felt slight difficulty in removing it, and immediately loosened the forceps. The intestine then prolapsed through the cervical ostium into the vagina (Figure 1(a)).

**Figure 2:**
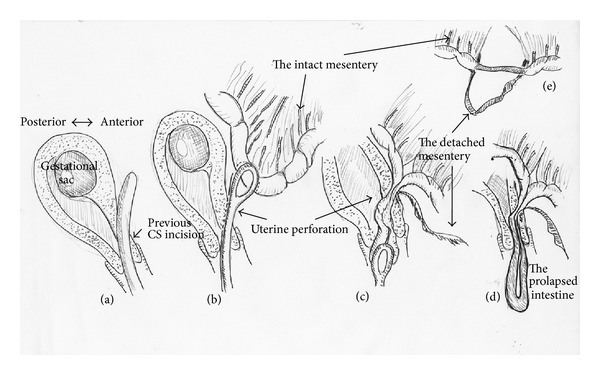
Schematic of the event. (a) The anterior uterine body wall was perforated with the Hegar dilator. The previous cesarean section incision was normal. (b) The forceps were extracted from the uterus through the perforation, grasping the ileum. (c) The ileum was pulled through the perforation site. The mesentery was detached when the ileum was pulled through the narrow perforation site. (d) The ileum prolapsed into the vagina. The ileum was strangulated at the perforation site. (e) The mesentery was completely separated from the bowel, resulting in ileal necrosis. Strangulation at the site of narrow perforation hole (d) may also have caused ileal necrosis.

We immediately performed laparotomy. At 50 cm oral from the ileocecal junction, about 50 cm of the ileum was prolapsed through the uterine perforation (Figure 1(b)). The uterus was perforated at the anterior, lower part of the uterine body (Figure 1(b)). The perforation was of approximately 1.0 cm in diameter and oblique to the uterine wall, coinciding with Hegar's size and its insertion direction. The previous cesarean incision site appeared normal. No intestinal excrement was observed in the abdominal cavity. The intestine including the prolapsed ileum and other pelvic organs showed no injury. Interestingly, the mesentery was completely detached from the prolapsed ileum about 50 cm (Figures 1(c) and 1(d)). The prolapsed ileum, at the area of the detached mesentery, was necrotic. The prolapsed necrotic ileum (50 cm) was resected (Figure 1(d)) and then the intestine was reconstructed by ileoileal anastomosis. D&C was performed under ultrasound guidance. Since the uterine perforation site may have been infected, we opened the perforation, disinfected the area, and reconstructed the uterine wall with 2 layered sutures. Postoperative course was uneventful.  Figure 2 illustrates the patient's clinical course.

## 3. Discussion

Uterine perforation may occur more frequently than previously expected. Kaali et al. [2] found that uterine perforation occurred in 14/706 first-trimester elective abortion (1.98%), of which 12 were recognized only by laparoscopy immediately after abortion. All were successfully treated with conservative management [2]. Thus, many cases of perforation may go undetected and, when observed, can be treated without surgical intervention. However, perforation can accompany intestinal injuries, which require surgery.

Some previous reports described a case in which the small intestine [3, 4] or appendix [5] entered the uterine cavity through a uterine perforation, namely, incarcerated bowels. Its extreme outcome is the intestinal prolapse as described here. Recently, Augustin et al. [6] reviewed the D&C-related bowel injury (not confined to intestinal prolapse). According to them, during the past 50 years, 10 case reports described 12 abortion-related intestinal prolapses [6]. The site of perforation was the uterine fundus (6 cases), posterior (2 cases), fundal anterior (1 case), or not described (3 cases). All 12 received intestinal resection and anastomosis, with the length of resected intestine being median of 200 cm (range 30–400). Two patients died. In the present patient, the anterior uterine wall was perforated and the length of resected intestine (50 cm) was shorter than the median of 200 cm, of which clinical significance is not clear due to the small number of reported cases.

Of the 12 reported by Augustin et al. [6], circumstances surrounding the procedure were described in 10 cases; 5 were regarded as “unsafe” or “criminal” abortions. Case series from remote area of Nigeria [7] demonstrated that nonphysicians caused this complication in 6 out of 9 such cases. Thus, “unsafe” abortion performed by inexperienced caregivers is considered the cause. However, as experienced obstetricians in a tertiary center in a developed country, we also had a patient with the same complication.

We must consider certain aspects of the D&C procedure. D&C is performed in a “blind” manner, meaning that it depends on tactile feedback to the operator. Experience and skill of individual providers are essential for a safe D&C. In addition, the procedure must be “observable.” Appropriate ultrasound imaging guidance for D&C is shown as follows.

1. Make a correct scanning plane to visualize the sound within the uterus.
2. Confirm that the sound reaches the gestational sac.
3. Maintain this scanning direction.
4. Insert a dilator under ultrasound visualization.
5. Insert the forceps to hold the product of conception under ultrasound guidance.

If ultrasound is available, it can be very useful. However, the nature of the procedure itself prevents complete avoidance of adverse events. Complications will occur in a certain percentage of patients even if the procedure is expected to be “routine” and performed by experienced obstetricians, as was the present case.

In the present patient, the mesentery may have been detached from the intestine when the intestine was pulled through the narrow perforation, which interrupted its blood supply, resulting in intestinal necrosis. Strangulation at the site of a narrow perforation may also have caused ileal necrosis. Necrotic intestine will almost surely perforate, leading to abdominal contamination, peritonitis, and systemic sepsis. If uterine perforation with bowel injury occurs, immediate laparotomy should be performed.

Although this complication is known among obstetricians, few cases have been reported. Thus, its nature and clinical characteristics have not yet been well established. Previous reports identified some risk factors of uterine perforation: the training level of the caregivers, advanced maternal age, greater parity, retroverted uterus, and history of prior abortion or cesarean section [6]. Only a small percentage of women with perforation suffer intestinal prolapse. Although the training level of the caregivers may also be a risk factor of intestinal prolapse [7], it is not known whether intestinal prolapse is caused by chance or some other risk factors. Augustin et al. [6] stated “we recommended publishing of every case on the subject for construction of more precise diagnostic and therapeutic algorithm,” with which we agree. This may allow for the future establishment of guidelines for dealing with this condition.

A “safe” abortion is “safe” only after its completion. We must make efforts to reduce the incidence of “unsafe” abortion regardless of the level of medical services available. Appropriate training and the use of ultrasound may reduce the number of “unsafe” abortions but we must also bear in mind that we cannot eliminate adverse events and, ultimately, there is no “routine” D&C.
